# LESS HOSPICE UTILIZATION AMONG CONNECTICUT MEDICAID DECEDENTS WITH NURSING HOME STAYS BEFORE DEATH

**DOI:** 10.1093/geroni/igad104.0246

**Published:** 2023-12-21

**Authors:** Ellis Dillon, Deborah Migneault, Noreen Shugrue, Dorothy Wakefield, Chae Man Lee, Doreek Charles, Bradley Richards, Julie Robison

**Affiliations:** UConn Health, Farmington, Connecticut, United States; UCONN Health, Farmington, Connecticut, United States; University of Connecticut, Farmington, Connecticut, United States; University of Connecticut, Farmington, Connecticut, United States; Univ of CT Health, Farmington, Connecticut, United States; University of Connecticut, Farmington, Connecticut, United States; State of Connecticut, Hartford, Connecticut, United States; UConn Health, Center on Aging, Farmington, Connecticut, United States

## Abstract

Hospice use, and length of enrollment of over 7 days are both considered indicators of quality care at the end of life, but the potential impact of nursing home (NH) stays on these outcomes is not well understood. This study analyzes hospice use and short hospice enrollment (0-7 days) for decedents in Connecticut’s Medicaid program who died between 2017 and 2020 and had a hospice-appropriate diagnosis. We compare individuals who had a NH stay within 60 days of their death to those who did not. Among 26,264 decedents meeting inclusion criteria, 14,227 (54.2%) had a NH stay within 60 days of death. Overall, 12,368 (47.1%) of all decedents received hospice care prior to death. Patients with a NH stay within 60 days of death were less likely than those without one to use hospice (44.3% vs 50.4%, p<0.01) (OR=0.78, (95% CI: 0.74, 0.82), p<0.01). Among those receiving hospice care, 4911 (39.7%) had short hospice enrollment. Patients with a NH stay within 60 days of death were 2.4 times more likely to have short hospice enrollment (OR=2.41 (95% CI: 2.24, 2.60), p<0.01) compared to those without. These findings suggest a need for further research into why individuals with NH stays are less likely to use hospice care and more likely to have shorter hospice enrollment. Comparing Medicaid decedents with NH stays prior to death to those living in the community may reveal disparities and areas that would benefit from interventions or policy change.

